# Interactions between Cytosolic Phospholipase A2 Activation and Mitochondrial Reactive Oxygen Species Production in the Development of Ventilator-Induced Diaphragm Dysfunction

**DOI:** 10.1155/2019/2561929

**Published:** 2019-04-18

**Authors:** Xian-Long Zhou, Xiao-Jun Wei, Shao-Ping Li, Rui-Ning Liu, Ming-Xia Yu, Yan Zhao

**Affiliations:** ^1^Emergency Center, Zhongnan Hospital of Wuhan University, 169 Donghu Road, Wuhan, Hubei 430071, China; ^2^Department of Clinical Laboratory, Zhongnan Hospital of Wuhan University, 169 Donghu Road, Wuhan, Hubei 430071, China

## Abstract

Cytosolic phospholipase A2 (cPLA2) has been reported to be critical for infection-induced mitochondrial reactive oxygen species (ROS) production and diaphragm dysfunction (DD). In the present study, we aim to investigate whether cPLA2 was involved in ventilator-induced diaphragm dysfunction (VIDD). Our results showed that mechanical ventilation (MV) induced cPLA2 activation in the diaphragm with excessive mitochondrial ROS generation and muscle weakness. Specific inhibition of cPLA2 with CDIBA resulted in decreased mitochondrial ROS levels and improved diaphragm forces. In addition, mitochondria-targeted antioxidant MitoTEMPO attenuated ventilator-induced mitochondrial oxidative stress and downregulated cPLA2 activation *in vivo*. Both CDIBA and MitoTEMPO were able to attenuate protein degradation, muscle atrophy, and weakness following prolonged MV. Furthermore, laser Doppler imaging showed that MV decreased diaphragm tissue perfusion and induced subsequent hypoxia. An *in vitro* study also demonstrated a positive association between cPLA2 activation and mitochondrial ROS generation in C2C12 cells cultured under hypoxic condition. Collectively, our study showed that cPLA2 activation positively interacts with mitochondrial ROS generation in the development of VIDD, and ventilator-induced diaphragm hypoxia serves as a possible contributor to this positive feedback loop.

## 1. Introduction

Diaphragm dysfunction (DD) occurring following long-term mechanical ventilation (MV), which is widely known as ventilator-induced mechanical ventilation (VIDD), is a great concern in the modern era of MV. Several studies showed that controlled MV (CMV) leads to decreased diaphragmatic force-generating capacity in various animal models [1, 2]. *In vitro* measurements of diaphragm contractile properties suggest that the decrease in contractility is an early and progressive phenomenon [3]. Physiologically, CMV induces diaphragm atrophy as early as 12 hours after induction of MV, and the atrophy develops rapidly [4]. Numerous evidences suggested that, in addition to protein synthesis, proteolysis plays a dominant role in diaphragm atrophy after CMV [5]. In addition, the onset of oxidative injury is rapid and long-lasting in animal MV models and has been demonstrated to be a key upstream regulator of protease system activation that contributes to diaphragm muscle atrophy and weakness [6].

Currently, it has been commonly accepted that mitochondria are the major source of reactive oxygen species (ROS) generation that induces the activation of protease and subsequent diaphragm atrophy during MV [7]. A recent study also suggested that diaphragm mitochondrial ROS formation, which mediated diaphragm weakness, may be critically dependent on PLA2 activation [8]. In addition, cPLA2 has been found to modulate cytokine-induced calpain activation in C2C12 cells and infection-induced diaphragm weakness in animals [9]. These results indicate that cPLA2 activation possibly induces acute oxidant injury and increases calpain activity, which finally results in DD. Moreover, a previous study showed that prolonged MV results in a time-dependent decrease in the ability of the diaphragm to augment blood flow to match O_2_ demand in response to contractile activity [10]. This study was attempting to speculate that the ventilator-induced decrease in diaphragmatic oxygenation could promote a hypoxia-induced generation of ROS in diaphragm muscle fibers and contribute to VIDD. However, whether cPLA2 activation contributes to oxidative stress and the development of VIDD has not yet been reported. In the present study, we aim to investigate whether cPLA2 activation contributes to ventilator-induced mitochondrial ROS generation and DD and to investigate the mechanism by which the ventilator induces cPLA2 activation.

## 2. Methods and Materials

### 2.1. Animals, Cell Line, and Reagents

Adult male Wistar rats (SPF level), weighing 450 to 550 g, were purchased from the Charles River Laboratories (Beijing, China). All animal studies were performed in the Bio-Safety Level III Laboratory of Wuhan University (Wuhan, Hubei, China).

All animals were kept in cages under controlled conditions (temperature: 25°C ± 2°C; relative humidity: 50%±5%) with a 12 : 12 light-dark cycle. Water and food were provided to the animals *ad libitum*. Animal experiments were performed in accordance with the Guidelines of Animal Care and Use. This study was approved by the Animal Experiment Center of Zhongnan Hospital of Wuhan University.

The C2C12 cell line was purchased from Procell (#CL-0044, Wuhan, Hubei, China). C2C12 cells were cultured in Dulbecco's modified Eagle medium (DMEM; Invitrogen, Carlsbad, CA) supplemented with 10% fetal bovine serum (FBS) and penicillin/streptomycin at 37°C and 5% CO_2_ in a humidified chamber.

Pentobarbital sodium was purchased from AMRESCO (Cleveland, OH, USA). Krebs-Henseleit bicarbonate buffer was purchased from M&C Gene Technology Ltd. (Beijing, China). The cPLA2 activity assay kit was purchased from Cayman Chemical (765021-96T, MI, USA). A MitoSOX Red mitochondrial superoxide indicator was purchased from Yaesen (#40778ES50, Shanghai, China). A Hydrogen Peroxide Colorimetric/Fluorometric Assay Kit was purchased from BioVision (Palo Alto, CA, USA). AACOCF3 was purchased from APEXBIO (B6748, Shanghai, China). SS-31 was purchased from Chinapeptides (Shanghai, China). MitoTEMPO was purchased from Enzo Life Sciences (NY, USA). CDIBA was purchased from Axon Medchem (VA, USA). Primary antibodies including anti-cPLA2, anti-p-cPLA2, anti-Atrogin-1, anti-MuRF1, anti-HIF-1*α*, anti-Slow Skeletal Myosin Heavy Chain (NOQ7.5.4D), anti-Fast Myosin Skeletal Heavy Chain (MY-32), and anti-Laminin were purchased from Abcam (Shanghai, China).

### 2.2. Study Protocol

#### 2.2.1. Animal Study

To investigate whether prolonged MV induces cPLA2 activation in the diaphragm and the roles of cPLA2 activation in ventilator-induced ROS generation and diaphragm muscle weakness, animals were assigned to the following groups: (1) control group (*n* = 5): animals received sham operation without ventilation; (2) mechanical ventilation group (PMV, *n* = 5): animals received MV for 12 hours; (3) MV+CDIBA group (*n* = 5): animals received MV for 12 hours with a single injection of CDIBA (a cPLA2 inhibitor, intraperitoneally (IP), 30 mg/kg^−1^ body weight) at the onset of ventilation; then, a continuous intravenous injection of CDIBA (5 mg/kg^−1^/h^−1^) was given throughout this study; (4) MV+MitoT group (*n* = 5): animals received MV for 12 hours with a single injection of MitoTEMPO (a mitochondria-targeted antioxidant, IP, 30 mg/kg^−1^) at the onset of ventilation; then, an intravenous injection of MitoTEMPO (10 mg/kg^−1^/h^−1^) was given during experiment; and (5) MV+CDIBA+MitoT group (*n* = 5): animals received MV for 12 hours with both CDIBA and MitoTEMPO treatments.

#### 2.2.2. Cell Study

Cells were seeded into four-well rectangular plates, the surface of which was coated with Matrigel (Becton, Dickinson and Co., Franklin Lakes, NJ, USA), at a density of 2.5 × 10^5^ cells/well, with 3 mL of DMEM (25 mM glucose; Invitrogen, Carlsbad, CA, USA) supplemented with 10% FBS and 1% penicillin-streptomycin. The cells were maintained in an incubator at 37°C under a 5% CO_2_ atmosphere. Then, cells were cultured under hypoxia for 0, 2, 4, 8, and 12 hours to create an ex vivo hypoxia model. In a parallel study, C2C12 cells cultured under hypoxia for 12 hours with cPLA2-specific inhibitor AACOCF3 (15 *μ*M) or mitochondria-targeted antioxidant SS31 (10 *μ*g/mL). At the end of hypoxia, western blot assay was performed for the measurements of p-cPLA2, cPLA2, and HIF-1*α*. Cell mitochondrial ROS levels were analyzed using a MitoSOX Red mitochondrial superoxide indicator. The cPLA2 and calpain activities were also measured.

### 2.3. *In Vitro* Hypoxia Model

A cell hypoxia model was established using a modular incubator chamber as previously reported [11]. In brief, cell cultures are placed in the hypoxic chamber. At the same time, a Petri dish containing sterile water has also been placed in the chamber to provide adequate humidification. The “twin” cell culture in normoxia is prepared as a control. Then, the chamber is closed to create hypoxia, and tubing is attached to a “hypoxia tank” containing a 1% O_2_ gas mixture. Finally, the chamber is returned to a conventional incubator for the desired period of time.

### 2.4. Mechanical Ventilation

An animal mechanical ventilation model was established and modified as previously described [10]. In detail, animals were anesthetized with sodium pentobarbital (40 mg/kg body weight, IP). After anesthesia, animals were placed on a recirculating heating blanket and fixed. Then, animals were tracheostomized and connected to a volume-driven small animal ventilator (VentElite, Harvard Apparatus; Cambridge, MA, USA). The tidal volume (TV) was set at 5 mL/kg body weight, and the respiratory rate (RR) was set at 55 to 60 breaths/min. Breathing air was humidified and enriched with oxygen. To avoid ventilator-induced systemic hypoxia, the ventilator parameters and oxygen flow were adjusted to maintain PaO_2_ between 80 and 100 mmHg and PaCO_2_ between 35 and 45 mmHg during the entire study. The values of PaO_2_ and PaCO_2_ were determined through arterial blood gas analysis, which was performed every 2 hours during the study. Blood pressure (BP) and heart rate (HR) were monitored at the tail using tail cuff plethysmography (BP-2010 Series Blood Pressure Meter, Softron, Japan). The right jugular vein was cannulated for continuous infusion of normal saline (Baxter, Deerfield, IL) at a rate of 1 mL/kg^−1^/h^−1^ and pentobarbital sodium (10 mg/kg^−1^/h^−1^) using an electric pump. Body temperature was maintained at 37°C during the experiment by external warming with a homeothermic blanket system.

### 2.5. Laser Doppler

Diaphragm muscle blood flow was measured using laser Doppler (PeriFlux System 5000). The diaphragm was exposed by midline laparotomy, and the probe of the laser Doppler was applied perpendicularly against the dorsal part of the diaphragm. The soleus was exposed after careful dissection of the right leg. The probe was then applied perpendicularly against the soleus muscle. Diaphragmatic signal analysis was restricted to the end-expiratory phase to avoid any influence of diaphragm motion on the recordings. The information is picked up by the returning fiber and displayed as perfusion units (PU).

### 2.6. Blood Sample Analysis

Arterial blood samples were collected every 2 hours for blood gas analysis during mechanical ventilation. Ventilator settings were adjusted according to blood gas results to maintain appropriate PaO_2_ and PaCO_2_. Blood gas analysis was performed using an i-STAT1 Analyzer (Abbott, Kyoto, Japan). Blood cell counts were performed using an automatic blood cell analyzer *Pentra MS CRP* (HORIBA Medical, Kyoto, Japan).

### 2.7. Cytosolic Phospholipase A2 Activity Assay

cPLA2 activities were determined as previously described [8]. In detail, tissue and cell samples were prepared by homogenization in buffer and subsequent protein determination of homogenates. Samples, blanks, and a positive control were loaded on a 96-well plate. Then, substrates were added into each well and incubated in the dark for 1 hour. The DTNB mixture (10 *μ*L) was added and well mixed. After 5 minutes, optical density was read at 414 nm and activity was calculated.

### 2.8. Calpain Activity Assay

Calpain activities were determined using a commercial kit (Abcam) according to the manufacturer's instructions. In brief, tissue and cell samples were prepared by homogenization in buffer and subsequent protein determination of homogenates. Then, samples, and positive and negative controls, were added into a 96-well plate. Then, the 10x reaction buffer and calpain substrate were added into each well and incubated in the dark for 30 minutes at 37°C. Fluorescence at Ex 400 nm/Em 505 nm was measured.

### 2.9. Measurements of Muscle Contractile Properties

Each muscle strip was rapidly mounted in a tissue chamber containing Krebs-Henseleit (K-H) solution. The solution was bubbled with a gas mixture of 95% O_2_-5% CO_2_ and maintained at 27°C and pH 7.4. Muscle extremities were held in spring clips and attached to an electromagnetic force transducer. Diaphragm muscle strips were electrically stimulated in twitch and tetanus with two silver electrodes positioned parallel to the muscle and delivering electrical stimulation lasting 1 ms. Muscle strips recovered their optimal mechanical performance after a 20-minute equilibration period. Strips were stimulated in twitch and then sequentially in tetanus with trains of 10, 20, 40, 60, 80, 100, and 120 Hz stimuli, and force and shortening were recorded. Fatigability was assessed by means of 330 ms stimulations repeated at 25 Hz and applied every second over a 5-minute period. Forces were measured at 2 minutes after stimulation, and the fatigue index (FI) was calculated as force at the onset of stimulation (*F*_0_)/force at 2 minutes after stimulation (*F*_2min_). At the end of the experiment, each muscle cross-sectional area (in cm^2^) was calculated from the ratio of muscle weight to muscle length at *L*_max_, assuming a muscle density of 1.06. All analyses were performed from digital records of force and length obtained with a computer.

### 2.10. Detection of Cellular Mitochondrial ROS Production

Mitochondrial ROS levels in cells were measured using a MitoSOX Red mitochondrial superoxide indicator. DMSO 13 *μ*L was added into 50 *μ*g of MitoSOX Red mitochondrial superoxide indicator and mixed well. MitoSOX Red mitochondrial superoxide indicator (5 mM) was diluted with Hank's balanced salt solution (HBSS) buffer into a final concentration of 5 *μ*M (working solution). Cells were soaked on a cover glass with 1 to 2 mL of working solution and cultured for 10 minutes under 37°C. Cells were washed with prepared buffers three times. Staining and observation were conducted under a microscope.

### 2.11. Detection of Diaphragm Mitochondrial H_2_O_2_ Generation

Mitochondrial isolation was performed as previously described [12]. Then, mitochondrial H_2_O_2_ levels were detected using a commercial Hydrogen Peroxide Assay Kit (BioVision Inc., CA, USA) according to the manufacturer's instruction. The absorbance was determined at 570 nm.

### 2.12. Western Blotting

Proteins were extracted from tissue samples using a protein extraction reagent (Sigma, USA), following a protocol provided by the manufacturer. Cells were lysed in radioimmunoprecipitation assay (RIPA; P0013B, Beyotime Biotechnology, Shanghai, China), and protein concentrations were determined using the BCA protein assay kit (Beyotime, Shanghai, China). Equal amounts of proteins were resolved by sodium dodecyl sulfate polyacrylamide gel electrophoresis (SDS-PAGE), and the proteins were transferred to Hybond ECL membranes (Amersham, Buckinghamshire, UK). The membranes were incubated with primary antibodies including anti-cPLA2, anti-p-cPLA2, anti-Atrogin-1, anti-MuRF-1, and anti-HIF-1*α* at 4°C overnight. After washing with TBST, the membranes were probed with HRP-labeled secondary antibodies. The membranes were visualized using an enhanced chemiluminescence system (Kodak, Rochester, NY, USA). Glyceraldehyde-3-phosphate dehydrogenase (GAPDH) was used as a loading control.

### 2.13. Immunofluorescence Staining

In the present study, immunofluorescence staining was performed in a blinded manner on paraffin-embedded tissues to evaluate the cross-sectional area of muscle fibers as previously described [13]. NOQ7.5.4D antibody was used for the identification of the slow myosin heavy chain, and the MY-32 antibody was used for the fast myosin heavy chains.

### 2.14. Statistical Analysis

Data are expressed as mean ± standard deviation (SD). Between-group comparisons were performed with one-way or two-way repeated measures analysis of variance followed, when significant, by a post hoc Tukey test for normally distributed data (Kolmogorov-Smirnov test) and a Kruskal-Wallis test followed by the Dunn post hoc test for nonnormally distributed data [14]. All statistical analyses were performed using GraphPad 5 (GraphPad Software, La Jolla, CA). All *p* values were two-tailed, and a *p* value less than 0.05 was considered significant.

## 3. Results

### 3.1. Systemic Response of Animals to MV

Arterial blood pressure, heart rate, and body temperature were monitored throughout the experiment, and no significant differences were observed between ventilation groups at the end of study (*p* > 0.05, respectively) (sTable 1). Blood gases and lactate levels are summarized in Table 1. Differences in pH values, bicarbonate, PaO_2_, and PaCO_2_ levels were insignificant between groups. In addition, there was no significant difference in lactate levels between all experimental groups (*p* > 0.05, respectively). As shown in Table 2, blood cell counts had slight increases in leukocytes and neutrophils in the MV, MV+CDIBA, MV+MitoT, and MV+CDIBA+MitoT groups compared with controls. However, the differences were insignificant (*p* > 0.05, respectively). Together, MV did not induce acid-base imbalance and systemic infections in animals in the present study.

### 3.2. Positive Interactions between cPLA2 Activation and Mitochondrial ROS Production in Diaphragm following MV

**Figure 1:**
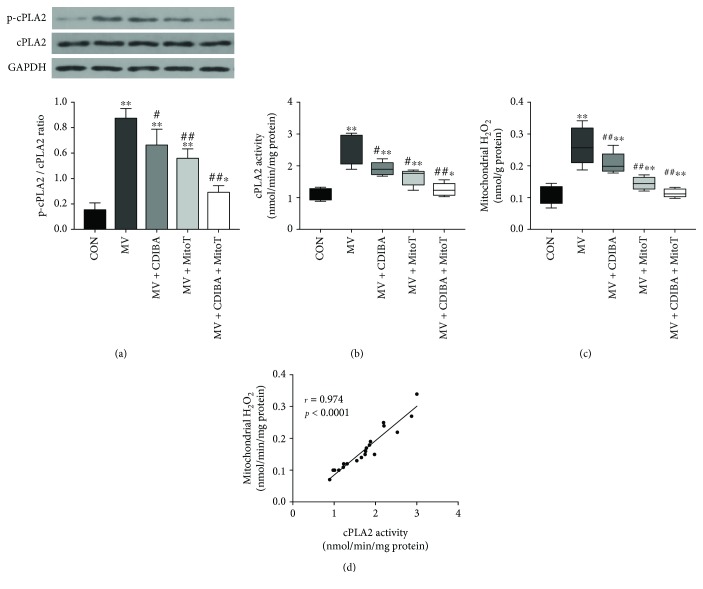
Interactions between cPLA2 and mitochondrial ROS generation in the diaphragm following MV. (a) Western blots for cPLA2 and p-cPLA2 in the diaphragm; (b) cPLA2 activity assay; (c) mitochondrial H_2_O_2_ generation in the diaphragm; (d) correlation analysis between cPLA2 activities and mitochondrial H_2_O_2_ levels. MV = mechanical ventilation; cPLA2 = cytosolic phospholipase A2; CDIBA = 4-{2-[5-chloro-1-(diphenyl-methyl)-2-methyl-1H-indol-3-yl]-ethoxy} benzoic acid, a specific cPLA2 inhibitor; MitoTEMPO = a mitochondria-targeted antioxidant. ^∗^ *p* < 0.05 and ^∗∗^ *p* < 0.01 vs. the control group; ^#^ *p* < 0.05 and ^##^ *p* < 0.01 vs. the MV group.

Here, we aim to determine the effects of cPLA2 in MV-induced mitochondrial ROS generation in the diaphragm. As seen in Figures 1(a) and 1(b), cPLA2-specific inhibitor CDIBA significantly reduced cPLA2 activation in the rat diaphragm following 12 hours of MV. Interestingly, administration of mitochondria-targeted antioxidant MitoTEMPO also downregulated ventilator-induced cPLA2 activation in the diaphragm (*p* < 0.05, respectively). To investigate the effects of cPLA2 activation in mitochondrial ROS generation, we measured mitochondrial H_2_O_2_ production in the present study. Our results showed that mitochondrial H_2_O_2_ production in the diaphragm was significantly increased after 12 hours of MV. Importantly, mitochondrial H_2_O_2_ production was downregulated by the inhibition of cPLA2 activation with CDIBA (Figure 1(c)) (*p* < 0.05). Correlation analysis demonstrated a positive association between the cPLA2 activity and mitochondrial H_2_O_2_ generation levels (*r* = 0.974, *p* < 0.0001) (Figure 1(d)). Therefore, these results demonstrated a positive association between cPLA2 activation and mitochondrial ROS generation.

### 3.3. Inhibition of cPLA2 Activation and Mitochondria-Targeted Antioxidant Attenuated Ventilator-Induced Protein Degradation, Muscle Atrophy, and Weakness

**Figure 2:**
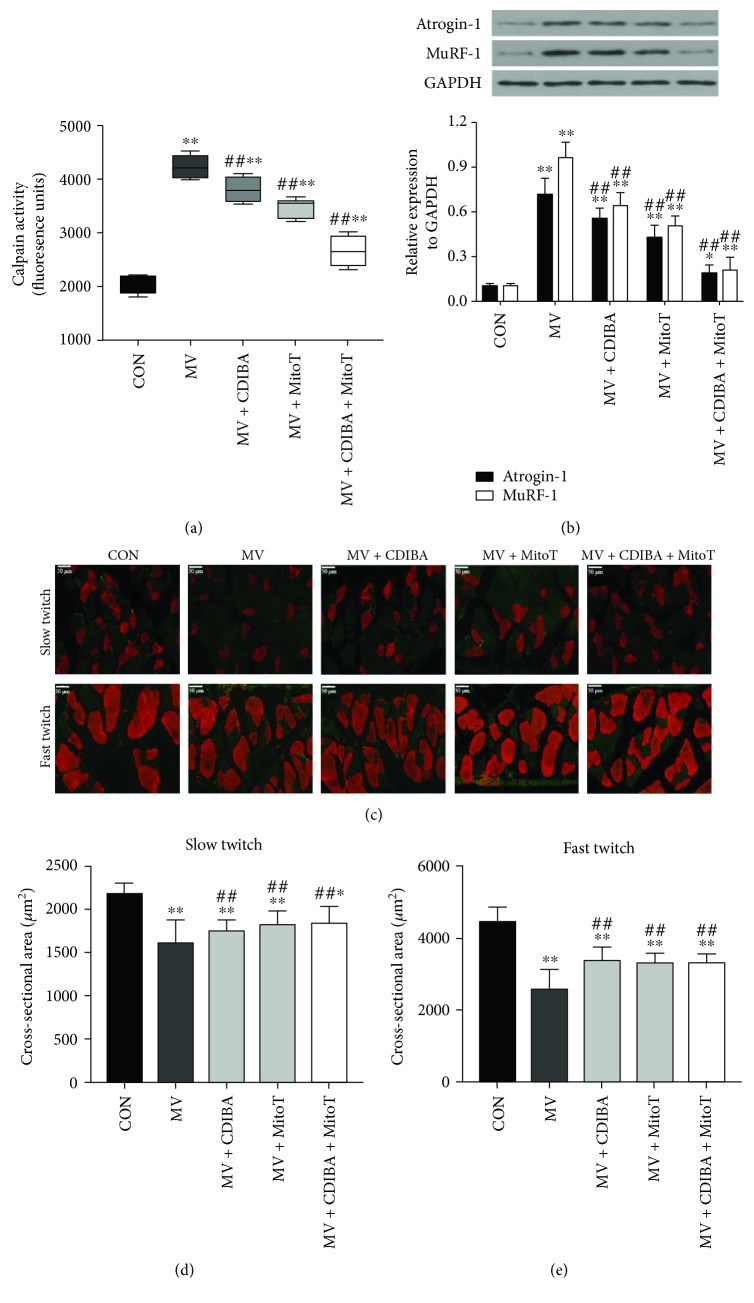
Protein degradation and atrophy of the diaphragm following MV. (a) calpain activity assay. (b) Western blots for Atrogin-1 and MuRF-1 expressions; (c) immunofluorescence staining for diaphragm muscle fibers; (d) cross-sectional area of slow twitch fibers; (e) cross-sectional area of fast twitch fibers. MV = mechanical ventilation; cPLA2 = cytosolic phospholipase A2; CDIBA = 4-{2-[5-chloro-1-(diphenylmethyl)-2-methyl-1H-indol-3-yl]-ethoxy} benzoic acid, a specific cPLA2 inhibitor; MitoTEMPO = a mitochondria-targeted antioxidant; ^∗^ *p* < 0.05 and ^∗∗^ *p* < 0.01 vs. the control group; ^##^ *p* < 0.01 vs. the MV group.

**Figure 3:**
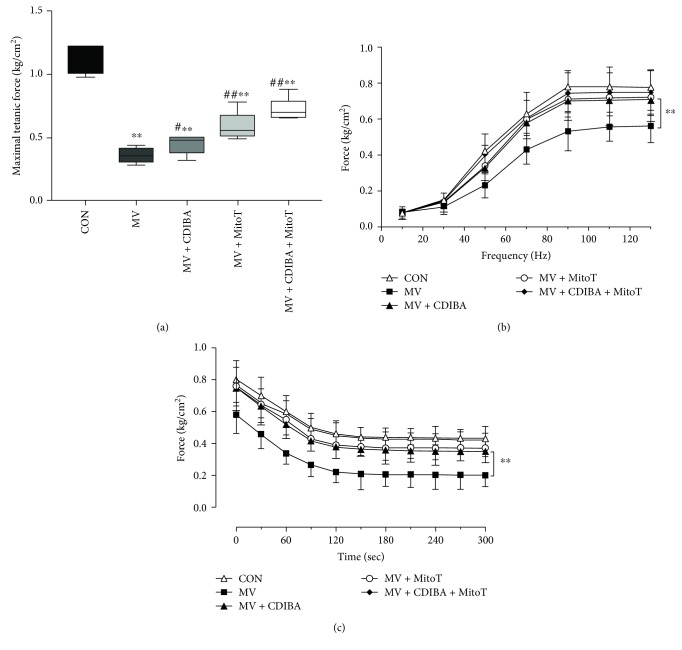
Diaphragm weakness after prolonged MV. (a) Maximal tetanic forces; (b) force-frequency curve; (c) fatigue tolerance test. MV = mechanical ventilation; cPLA2 = cytosolic phospholipase A2; CDIBA = 4-{2-[5-chloro-1-(diphenylmethyl)-2-methyl-1H-indol-3-yl]-ethoxy} benzoic acid, a specific cPLA2 inhibitor; MitoTEMPO = a mitochondria-targeted antioxidant. ^∗∗^ *p* < 0.01 vs. the control group; ^##^ *p* < 0.01 vs. the MV group.

Since mitochondrial ROS generation is the key regulator of proteolysis in the diaphragm following MV, proteolytic markers were determined for the evaluation of protein degradation. As seen in Figure 2(a), calpain activities were significantly increased in the MV group compared with controls. Specific inhibition of cPLA2 activation with CDIBA or mitochondria-targeted antioxidant MitoTEMPO significantly downregulated calpain activities in the diaphragm (*p* < 0.05, respectively). The combination of CDIBA and MitoTEMPO further decreased diaphragm calpain activities. Moreover, western blot analysis demonstrated similar changes in atrophy-related genes Atrogin-1 and MuRF-1 as that of calpain in the diaphragm following MV (Figure 2(b)). Immunofluorescence staining suggested that MV induced apparent muscle atrophy, as the CSA of diaphragm muscle fibers were significantly decreased after 12 hours of MV in all ventilated animals, whereas CDIBA and/or MitoTEMPO treatments increased CSA of muscle fibers (Figures 2(c) and 2(e)). Moreover, *in vitro* measurements of muscle strip contractile properties demonstrated significant decreases of muscle forces in animals that underwent MV. However, administration of either CDIBA or MitoTEMPO apparently attenuated muscle weakness with increased maximal tetanic forces, force-frequency curves, and fatigue tolerances compared with the MV group (Figure 3). Collectively, these data confirmed that cPLA2 activation mediated mitochondrial ROS generation and its subsequent events including protein degradation, muscle atrophy, and weakness.

### 3.4. Diaphragmatic Hypoxia Contributed to cPLA2 Activation and Mitochondrial ROS Generation

**Figure 4:**
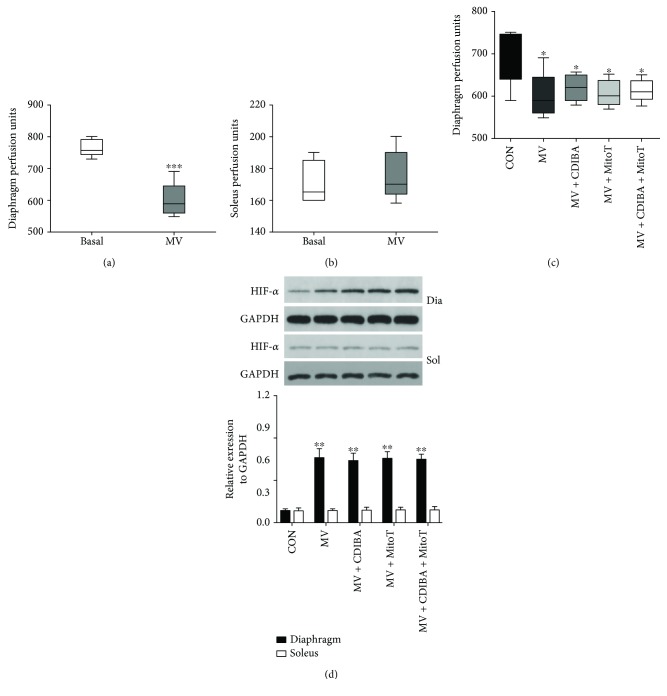
Ventilator decreased diaphragm perfusion. (a) Laser Doppler for diaphragm perfusion; (b) laser Doppler for soleus perfusion; (c) effects of CDIBA and MitoTEMPO on diaphragm perfusion after 12 hours of MV; (d) western blots for HIF-1*α* in the diaphragm and soleus. MV = mechanical ventilation; cPLA2 = cytosolic phospholipase A2; CDIBA = 4-{2-[5-chloro-1-(diphenylmethyl)-2-methyl-1H-indol-3-yl]-ethoxy} benzoic acid, a specific cPLA2 inhibitor; MitoTEMPO = a mitochondria-targeted antioxidant. ^∗^ *p* < 0.05 and ^∗∗^ *p* < 0.01 vs. the control group; ^∗∗∗^ *p* < 0.001 vs. basal.

**Figure 5:**
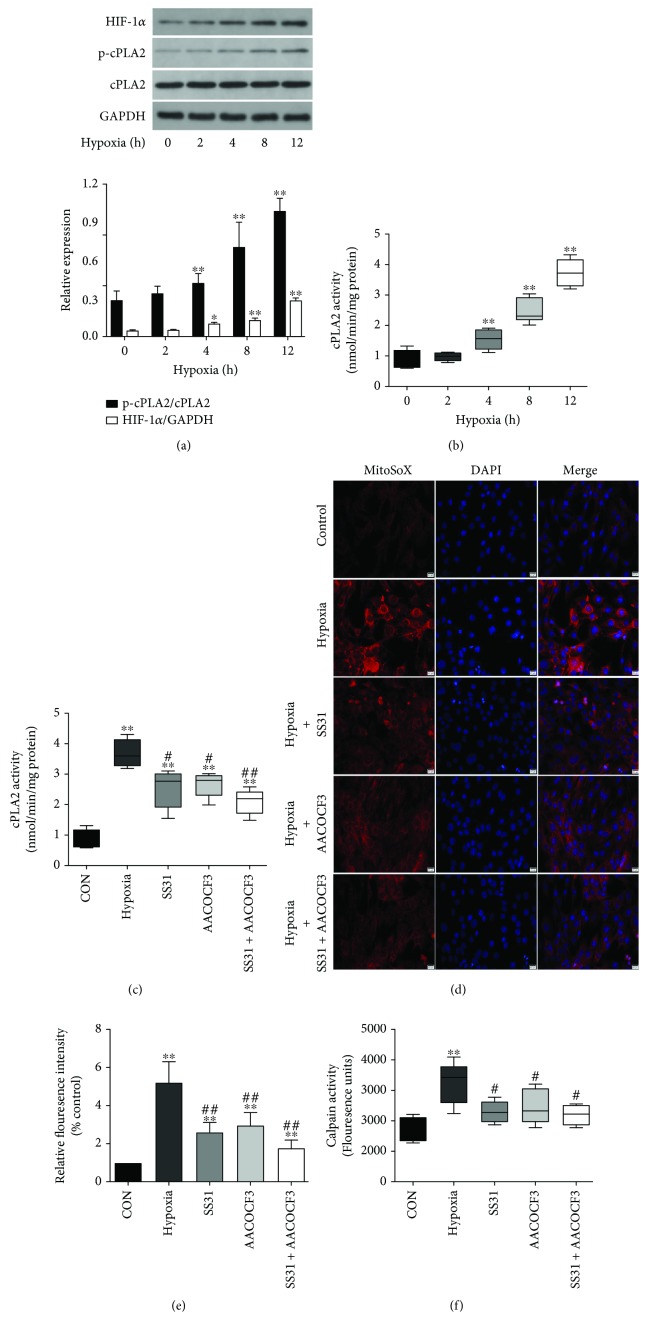
Hypoxia induced cPLA2 activation and mitochondrial ROS generation *in vitro*. (a) Western blots for HIF-1*α* expressions in C2C12 cells; (b) changes of cPLA2 activities in C2C12 cells underwent indicated time of hypoxia; (c) effects of AACOCF3 and SS31 on cPLA2 activities in C2C12 cells after 12 hours of hypoxia; (d) MitoSOX assay for the detection of mitochondrial ROS generation in C2C12 cells; (e) fluorescence intensities of MitoSOX; (f) effects of AACOCF3 and SS31 on calpain activities in C2C12 cells after 12 hours of hypoxia. AACOCF, a specific inhibitor of cPLA2; SS31, a mitochondria-targeted antioxidant. ^∗^ *p* < 0.05 and ^∗∗^ *p* < 0.01 vs. the control group; ^#^ *p* < 0.05 and ^##^ *p* < 0.01 vs. the MV group.

In this study, we used a laser Doppler machine and probes to determine the microvascular blood flow in the soleus and diaphragm following MV. As seen in Figure 4(a), the perfusion units (PU) were significantly decreased at the end of ventilation compared with the baseline in the MV (768 ± 26 vs. 600 ± 54 PU, *p* < 0.05) group. However, there were no significant changes in PU of the soleus in ventilated animals (Figure 4(b)). In addition, treatments with CDIBA and/or MitoTEMPO did not alter diaphragm tissue perfusion compared with the MV group (*p* > 0.05, respectively) (Figure 4(c)). Next, we checked whether MV induced tissue hypoxia in the diaphragm. Western blot analysis showed that MV for 12 hours induced apparent expression of HIF-1*α* in the diaphragm but not in the soleus (Figure 4(d)). These results suggest that prolonged MV induced hypoxia in the diaphragm but not in the soleus. Hypoxia, as a complex stress, is able to induce cellular oxidative stress and cPLA2 activation. Therefore, we performed an *in vitro* study to investigate the possible association between hypoxia and cPLA2 activation in C2C12 myoblast cells. As seen in Figures 5(a) and 5(b), hypoxia induced cPLA2 phosphorylation and increased cPLA2 activities in a time-dependent manner. In addition, administration of either cPLA2 inhibitor AACOCF3 or mitochondria-targeted antioxidant SS31 significantly downregulated hypoxia-induced cPLA2 activation *in vitro* (Figure 5(c)). Importantly, MitoSOX assays suggested that hypoxia was able to induce mitochondrial ROS generation *in vitro*, which can be also diminished by the treatment of AACOCF3 or SS31 (Figures 5(d) and 5(e)). Furthermore, hypoxia for 12 hours was able to induce calpain activation in C2C12 cells, which can also be downregulated by the treatment of AACOCF3 and/or SS31 (Figure 5(f)). Collectively, these results suggest that hypoxia serves as a possible contributor to the cPLA2 activation and mitochondrial ROS generation in the diaphragm after MV. In addition, this *in vitro* study also confirmed the positive interaction between cPLA2 activation and mitochondrial ROS generation.

## 4. Discussion

The major findings of this study can be summarized as follows: (1) prolonged MV for 12 hours is able to induce cPLA2 activation in the rat diaphragm; (2) ventilator-induced cPLA2 activation positively interacts with mitochondrial ROS generation, which promotes the development of VIDD; and (3) MV decreases diaphragm tissue perfusion and induces expression of HIF-1*α*, and diaphragm hypoxia possibly contributes to cPLA2 activation and mitochondrial ROS generation following MV.

The imbalance between protein synthesis and proteolysis occurred in the diaphragm following prolonged MV results in muscle atrophy and DD. It has been shown that the upstream regulatory signaling of these proteolytic events is linked to the mitochondrion-derived ROS production in the diaphragm [7, 15]. In addition, elevated mitochondrial oxidative stress (MOS) has been observed in both animal models and human samples [16]. Importantly, a previous study has demonstrated that MOS is sufficient to trigger the proteolytic process *in vitro* [15]. In the present study, we observed that prolonged MV increased mitochondrial H_2_O_2_ production and calpain activation in the diaphragm. The hypothesis that prolonged MV induced cPLA2 activation in the diaphragm has been confirmed in this study. Due to the key role of cPLA2 in mediating lipid mediator production and its widespread tissue expression, it has been implicated in regulating homeostatic processes and disease pathogenesis throughout all organ systems. Several studies have demonstrated the involvement of cPLA2 in diverse pathological processes including skeletal muscle atrophy. cPLA2*α* ^–/–^ mice are known to have increased striated muscle growth [17]. In addition, cPLA2 mediated fatty acid hydroperoxide generation by mitochondria in denervation-associated muscle atrophy [18]. The relation between the cPLA2 activation and diaphragm muscle atrophy may relate to the mitochondrial oxidative stress. A previous study suggested that the activation of phospholipase A2 is involved in indomethacin-induced damage in Caco-2 cells [19]. In that study, cytosolic phospholipase A2 activation was presented with the increases of oxidative stress in cells treated with indomethacin. Zhu et al. reported that the A*β*_1–42_-mediated increase in mitochondrial ROS production in astrocytes was suppressed by MAFP, a specific cPLA2 inhibitor [20]. In addition, cPLA2 is one of the constitutively active PLA2s that serve as important mediators for the release of polyunsaturated fatty acids, including arachidonic acid (AA) and docosahexaenoic acid (DHA) from membrane phospholipids [21, 22]. AA has been shown to induce ROS generation through activation of NADPH oxidases (NOXs), which may play a key role in the expression of heme oxygenase-1 (HO-1) [23]. In addition, DHA has been reported to trigger ROS generation and finally induce oxidative stress-induced growth inhibitor 1 (OSGIN1) expression in breast cancer cells [24]. Collectively, all these studies suggest that cPLA2 plays an important role in ROS production in different pathological processes. In our study, we observed that the cPLA2 activity was positively associated with the total and mitochondrial ROS production in the diaphragm. This indicated that cPLA2 promoted MV-induced oxidative stress in the diaphragm via inducing mitochondrion-derived ROS production.

Another important finding of this study is the positive feedback loop between cPLA2 activation and mitochondrial ROS production. The mitochondrial ROS generation in the diaphragm was dependent on the activation of cPLA2, whereas treatments with antioxidants also reduced cPLA2 activities. In addition, the cell study showed that exogenous H_2_O_2_ induced cPLA2 activation, and specific inhibition of mitochondrial ROS using SS31 reduced H_2_O_2_-induced activation of cPLA2. Oxidative stress, or ROS, has been reported to promote cPLA2 activation and expression. Cheng and others reported that in human tracheal smooth muscle cells (HTSMCs), cigarette smoke extract (CSE) induced NADPH oxidase activation leading to phosphorylation of p42/p44 MAPK, p38 MAPK, and JNK [25]. These reactions induced NF-kappa B and AP-1 activities, which were essential for CSE-induced cPLA2 gene expression. In addition, oxidative stress or ROS accumulation may increase the permeability of the cell membrane and induce cytosolic calcium accumulation [26]. Moreover, PI3K/MAPK signaling can be activated by oxidative stress under different conditions [27]. Importantly, both cytosolic calcium levels and MAPK phosphorylation are the most important regulators that mediate the activation of cPLA2. This positive feedback between the activation of cPLA2 and mitochondrial oxidative stress in the diaphragm during MV might play a very important role in the development of VIDD. Therefore, it is possible that interventions focusing on the blockage of this loop (e.g., a combination of mitochondria-targeted antioxidants and cPLA2 inhibitors) can impact the activation of proteolytic systems and the development of VIDD.

In this study, we first found that ventilator-induced hypoxia serves as a contributor of cPLA2 activation and mitochondrial ROS generation in the diaphragm. In fact, there is a very close association between oxidative stress and hypoxia. Hypoxia is known to elicit excess ROS production and thereby alter redox balance, and both short- and long-term hypoxic exposures have been shown to induce oxidative stress [28, 29]. The combined presence of inactivity and hypoxia is rarely observed in healthy individuals. Here, in addition to the inactivity of the diaphragm during MV, our results showed that prolonged MV decreased blood flow and induced diaphragm tissue hypoxia. Therefore, the combination of inactivity and hypoxia exists in the diaphragm during MV, and those two factors are both able to induce oxidative stress, which is responsible for the activation of proteolytic systems. Our results also showed that diaphragm hypoxia induced cPLA2 activation, which is involved in the development of mitochondrial oxidative stress. However, the mechanism by which hypoxia induced cPLA2 activation remains unclear.

## 5. Conclusion

Collectively, our findings indicated that mitochondrial ROS generation in the diaphragm following prolonged MV was dependent on the activation of cPLA2. On the other side, mitochondrial oxidative stress also promoted diaphragmatic cPLA2 activation. Therefore, the positive feedback loop between cPLA2 and mitochondrial ROS probably accelerates the development of VIDD.

## Figures and Tables

**Table 1:** Arterial blood gas analysis at the end of study (mean ± SD).

	Control (*n* = 5)	MV (*n* = 5)	MV+CDIBA (*n* = 5)	MV+MitoT (*n* = 5)	MV+CDIBA+MitoT (*n* = 5)
pH	7.40 ± 0.01	7.38 ± 0.03	7.39 ± 0.05	7.40 ± 0.05	7.38 ± 0.02
PaCO_2_ (mmHg)	42 ± 5	41 ± 5	39 ± 4	41 ± 6	39 ± 5
PaO_2_ (mmHg)	101 ± 11	98 ± 10	101 ± 16	102 ± 8	94 ± 12
HCO_3_^−^ (mM)	24.2 ± 4.1	23.9 ± 3.2	23.0 ± 4.3	22.6 ± 3.8	17.9 ± 4.7
BE	2.9 ± 2.0	2.1 ± 1.3	3.0 ± 2.3	2.5 ± 1.1	2.9 ± 2.4
Lactate (mM)	1.25 ± 0.22	1.87 ± 0.67	1.85 ± 0.24	1.65 ± 0.40	1.80 ± 0.69

MV = mechanical ventilation; CDIBA = 4-{2-[5-chloro-1-(diphenylmethyl)-2-methyl-1H-indol-3-yl]-ethoxy} benzoic acid (cPLA2 inhibitor), a cPLA2 specific inhibitor; MitoT = MitoTEMPO, a mitochondria-targeted antioxidant; BE = base excess.

**Table 2:** Blood cell counts at the end of the study (mean ± SD).

	Control (*n* = 5)	MV (*n* = 5)	MV+CDIBA (*n* = 5)	MV+MitoT (*n* = 5)	MV+CDIBA+MitoT (*n* = 5)
Erythrocytes (×10^12^/L)	5.64 ± 0.21	5.41 ± 0.33	5.39 ± 0.15	5.40 ± 0.05	5.30 ± 0.26
Hemoglobin (g/L)	132 ± 21	129 ± 25	139 ± 34	131 ± 26	129 ± 15
Leukocytes (×10^9^/L)	6.72 ± 0.52	6.95 ± 1.21	6.81 ± 0.67	6.92 ± 1.11	7.04 ± 0.57
Neutrophils (×10^9^/L)	3.21 ± 0.12	3.28 ± 0.89	3.30 ± 0.63	3.26 ± 0.18	3.29 ± 0.07
Platelets (×10^9^/L)	112 ± 12	119 ± 21	111 ± 11	118 ± 22	109 ± 12

MV = mechanical ventilation; CDIBA = 4-{2-[5-chloro-1-(diphenylmethyl)-2-methyl-1H-indol-3-yl]-ethoxy} benzoic acid (cPLA2 inhibitor), a cPLA2 specific inhibitor; MitoT = MitoTEMPO, a mitochondria-targeted antioxidant.

## Data Availability

The data used to support the findings of this study are available from the corresponding author upon request.

## Supplementary Materials

Supplementary Materials sTable 1: body weight, MAP, HR, and RT (mean ± SD). BW = body weight;MAP = mean arterial pressure;HR = heart rate;RT = rectal temperature; MV = mechanical ventilation;CDIBA = 4-{2-[5-chloro-1-(diphenylmethyl)-2-methyl-1H-indol3-yl]-ethoxy} benzoic acid (cPLA2 inhibitor): MitoT = MitoTEMPO. BW was determined before the experiment. Values of MAP, HR, and RT at the end of study are presented here.
