# Supplementary material for: Interactions between Cytosolic Phospholipase A2 Activation and Mitochondrial Reactive Oxygen Species Production in the Development of Ventilator-Induced Diaphragm Dysfunction
Source: Oxid Med Cell Longev. 2019 Apr 18;2019:2561929. doi: 10.1155/2019/2561929 (PMC6501131; doi:10.1155/2019/2561929)
Supplement: Supplementary Materials — sTable 1: body weight, MAP, HR, and RT (mean ± SD). BW = body weight;MAP = mean arterial pressure;HR = heart rate;RT = rectal temperature; MV = mechanical ventilation;CDIBA = 4-{2-[5-chloro-1-(diphenylmethyl)-2-methyl-1H-indol3-yl]-ethoxy} benzoic acid (cPLA2 inhibitor): MitoT = MitoTEMPO. BW was determined before the experiment. Values of MAP, HR, and RT at the end of study are presented here. [file 2561929.f1.pdf]

Supplementary materials

sTable 1. Body weight, MAP, HR, and RT (mean ± SD)

Group (n =5)	BW (g)	MAP (mmHg)	HR (bpm)	RT (℃)
Control	464 ± 48	142 ± 22	398 ± 38	37.2 ± 0.1
MV	455 ± 32	137 ± 27	402 ± 29	37.1 ± 0.2
MV + CDIBA	467 ± 52	138 ± 13	387 ± 31	37.0 ± 0.2
MV + MitoT	458 ± 34	140 ± 19	399 ± 20	37.2 ± 0.3
MV + CDIBA + MitoT	472 ± 27	138 ± 25	407 ± 32	37.2 ± 0.3

BW = body weight；MAP = mean arterial pressure；HR = heart rate；RT = rectal temperature; MV = mechanical ventilation； CDIBA = 4-{2-[5-chloro-1-(diphenylmethyl)-2-methyl-1H-indol-3-yl]-ethoxy} benzoic acid (cPLA2 inhibitor): MitoT = MitoTEMPO. BW were determined before the experiment. Values of MAP, HR, and RT at the end of study are presented here.
